# Impact of High Fat Diet and Ethanol Consumption on Neurocircuitry Regulating Emotional Processing and Metabolic Function

**DOI:** 10.3389/fnbeh.2020.601111

**Published:** 2021-01-26

**Authors:** Caitlin R. Coker, Bailey N. Keller, Amy C. Arnold, Yuval Silberman

**Affiliations:** ^1^Biochemistry and Molecular & Cellular Biology, Georgetown University School of Medicine, Washington, DC, United States; ^2^Neural and Behavioral Sciences, Penn State College of Medicine, Hershey, PA, United States

**Keywords:** obesity, binge, alcohol use disorder (AUD), mental health, neuroinflammation

## Abstract

The prevalence of psychiatry disorders such as anxiety and depression has steadily increased in recent years in the United States. This increased risk for anxiety and depression is associated with excess weight gain, which is often due to over-consumption of western diets that are typically high in fat, as well as with binge eating disorders, which often overlap with overweight and obesity outcomes. This finding suggests that diet, particularly diets high in fat, may have important consequences on the neurocircuitry regulating emotional processing as well as metabolic functions. Depression and anxiety disorders are also often comorbid with alcohol and substance use disorders. It is well-characterized that many of the neurocircuits that become dysregulated by overconsumption of high fat foods are also involved in drug and alcohol use disorders, suggesting overlapping central dysfunction may be involved. Emerging preclinical data suggest that high fat diets may be an important contributor to increased susceptibility of binge drug and ethanol intake in animal models, suggesting diet could be an important aspect in the etiology of substance use disorders. Neuroinflammation in pivotal brain regions modulating metabolic function, food intake, and binge-like behaviors, such as the hypothalamus, mesolimbic dopamine circuits, and amygdala, may be a critical link between diet, ethanol, metabolic dysfunction, and neuropsychiatric conditions. This brief review will provide an overview of behavioral and physiological changes elicited by both diets high in fat and ethanol consumption, as well as some of their potential effects on neurocircuitry regulating emotional processing and metabolic function.

## Introduction

The prevalence of anxiety and depression has steadily increased in recent years in the United States, with over 18% of the population having anxiety (Kessler et al., 2005) and ~6.6% having depression each year (Kessler et al., 2003). Clinical studies show a correlation between poor diet and these conditions (Bonnet et al., 2005). Rates of overweight and obesity, often due to over-consumption of high fat diets (HFD) in western culture, are also rising in the United States and are associated with an increased risk for developing psychiatric conditions such as anxiety (Petry et al., 2008; Gariepy et al., 2010) and depression (Petry et al., 2008; Preiss et al., 2013). Binge eating disorders, which can often overlap with overweight and obesity outcomes, also show comorbidity with depression and anxiety disorders (Citrome, 2019). Thus, diet can have a strong impact on mental health. Depression and anxiety disorders are also often comorbid with alcohol (EtOH) and substance use disorders, and these interactions have been well-studied in both clinical (Kushner et al., 2000; Kingston et al., 2017) and preclinical (Pandey et al., 2005; Crews et al., 2016) research. Studies of the commonalities between binge eating disorders, overweight and obesity, and alcohol use disorders (AUD) suggest many overlapping neurological mechanisms may be involved (Rapaka et al., 2008). This brief review will discuss some of the predominant behavioral and physiological changes caused by HFD, EtOH, and the combination of the two as well as potential central mechanisms that may contribute to disruptions in emotional regulation and metabolic function. As these are very complex fields, we sought to highlight portions of the literature that may be most relevant to combined HFD and EtOH use and provided citations for further in-depth analysis when possible for topics outside the main scope of this review.

## Overlap of Clinical Outcomes Between Obesity and Alcoholism

Overweight and obesity are measured by a person's body mass index (BMI), which is calculated using weight and height. Although not a measure of overall health status, in general a BMI of 25–29.9 kg/m^2^ is considered overweight, while a BMI of ≥30 kg/m^2^ is defined as obese^1^. Overweight- and obesity-related conditions are the second leading cause of preventable death in the United States, attributing to 300,000 deaths yearly (Allison et al., 1999) and having an estimated $147 billion in medical costs annually^2^. The National Health and Nutrition Examination Survey reported the prevalence of obesity among adults in the United States was 42.4% in 2017–2018, an 11.9% increase from 1999 to 2000 (Hales et al., 2017). Importantly, obesity is a primary risk factor for an array of chronic diseases including cardiovascular disease, type II diabetes, hypertension, and certain cancers (Must and McKeown, 2000).

The National Institute on Alcohol Abuse and Alcoholism (NIAAA) defines AUD as “a chronic relapsing brain disease characterized by an impaired ability to stop or control EtOH use despite adverse social, occupational, or health consequences” (National Institute on Alcohol Abuse Alcoholism, 2020). The National Survey on Drug Use and Health (NSDUH) reported over 14 million adults had AUD in 2018^3^. This is a global health issue as The Global Status Report on Alcohol and Health reported an estimated 3 million EtOH-related deaths in 2016, which made up over 5% of all deaths worldwide. Of this, over 2.3 million deaths were among men (World Health Organization, 2018). The higher level of EtOH-related deaths in men may be due to the differing prevalence of AUD between men (237 million) and women (46 million), with the highest prevalence in the European region and Americas (World Health Organization, 2018) according to the World Health Organization. Overall, EtOH misuse is the third leading cause of preventable death in the United States and attributes to over 88 thousand deaths each year (Centers for Disease Control Prevention, 2020). In 2010, excessive EtOH drinking cost the United States $249 billion, with nearly 77% attributed to binge drinking (Sacks et al., 2015). Further, chronic EtOH misuse contributes to an estimated 200 diseases and injury-related conditions. According to the World Health Organization, of the 3 million EtOH-attributable deaths in 2016, 21.3% were due to digestive diseases, 19% due to cardiovascular diseases and diabetes, 12.9% due to infectious diseases, and 12.6% due to cancers (World Health Organization, 2018). In the United States, nearly 48% of all liver cirrhosis deaths were EtOH related in 2013 (Yoon and Chen, 2016).

Not only is the prevalence of obesity and AUD on the rise separately, there is also an emerging link between developing both obesity and AUD in the United States (Petry et al., 2008; Grucza et al., 2010). Clinical data indicate chronic and excessive EtOH drinking results in an increased risk for developing metabolic dysfunction (Fan et al., 2006) and type II diabetes (Kao et al., 2001; Carlsson et al., 2003), outcomes similar to that seen with overconsumption of HFD. The shared clinical consequences of both HFD and EtOH overconsumption suggest an overlap in the mechanisms by which these insults modulate insulin action and glucose homeostasis. Furthermore, the common mental health conditions separately associated with chronic HFD and EtOH overconsumption, i.e., depression and anxiety-related disorders, suggests overlap of central mechanisms of emotional regulation that may become dysregulated by HFD and chronic EtOH use.

## Models of High Fat Diet and Alcohol Intake

### High Fat Diet Exposure in Rodents as a Model of Obesity

Obesity in the clinical population can be attributed to both genetic and environmental factors, which can include a sedentary lifestyle and consumption of diets rich in carbohydrates and saturated fats. Rodent models involving diet manipulation can recapitulate the pathophysiology associated with diet-induced disease development in the absence or presence of predetermined genetic alterations to assess metabolic outcomes in whole-animal systems. These diet-induced obesity (DIO) models are commonly used in the preclinical setting to study the whole-body insults that occur during the progression of obesity. Rodent models of DIO have been used since the 1940s when researchers administered a highly palatable liquid diet *ad libitum* to stimulate weight gain in rats to the point of obesity (Ingle, 1949). Diets high in fat, sugar, and other nutrients have all been used in rodent models of DIO, with researchers incorporating ingredients such as Crisco (Mickelsen et al., 1955; Sclafani and Springer, 1976), chocolate (Sclafani and Springer, 1976; Burokas et al., 2018), and sucrose (Levin and Dunn-Meynell, 2002; Harzallah et al., 2016; Collins et al., 2018). This allows for comparison of the various diets found in the human population (e.g., western diets, cafeteria diets). Overall, the physiological mechanisms behind DIO models include hyperphagia of calorically dense diets, increased efficacy of dietary fat being stored in the body, a pre-diabetic phenotype (e.g., mild-modest hyperglycemia, hyperinsulinemia, insulin resistance, glucose intolerance) and alterations in the hormones involved in energy balance (Hariri and Thibault, 2010). This review will primarily focus on DIO produced by HFD feeding, as this is one of the most widely studied rodent models of obesity, particularly in terms of interactions with alcohol as well as dietary effects on depression- and anxiety-like behaviors. It is recognized, however, that some studies utilize other dietary models (e.g., high fat plus high sugar or high fructose/sucrose) as well as genetically- and pharmacologically-induced models of obesity to examine metabolic outcomes as well as measures of homeostatic and hedonic feeding behaviors as previously reviewed (Surwit et al., 1988; Pandit et al., 2012; Rosini et al., 2012; Stice et al., 2013; Hughey et al., 2014; Slomp et al., 2019). Since depression- and anxiety-like behaviors have been well-characterized as a risk factor for increased EtOH misuse, brain regions regulating these behaviors are an intriguing area of research on potential mechanisms of behavioral overlap of overconsumption of HFD and EtOH.

### Animal Models of Alcohol Intake

Various EtOH exposure methods have been studied in animal models for decades. In the 1960s, researchers sought to develop a clinically relevant animal model for EtOH consumption. Dr. Charles Lieber and Dr. Leonore DeCarli created a unique model by administering EtOH as part of a nutritionally complete liquid diet (Lieber and DeCarli, 1982). This consumption model has been the forerunner in the EtOH research field as a useful tool to study pathological disorders associated with AUD, such as alcoholic liver disease (Dolganiuc et al., 2009; Bhopale et al., 2017; Guo et al., 2018). Another commonly used exposure method is EtOH vapor inhalation which is useful to maintain constant, clinically relevant blood EtOH concentrations and to induce EtOH dependence (Healey et al., 2008; Snyder et al., 2019). In addition to these passive or forced consumption models, many rodent strains will self-administer to pharmacologically relevant blood EtOH concentrations. These self-administration models often utilize operant conditioning or two-bottle choice methods. The two-bottle choice model is a voluntary consumption model providing the animal a choice between a bottle containing an EtOH solution (typically in water, but other vehicles may be used) and a second bottle containing water (or vehicle). This model allows for behavioral analysis of intake including assessment of EtOH preference and consumption, typically in a home cage setting (Coker et al., 2020). Access schedules to the EtOH solution can be readily altered to induce binge-like intake (Melendez, 2011; Thiele et al., 2014) and allows for discrete time periods for access to EtOH and HFD. The C57BL/6 strain of mice is typically used for these experiments due to their consistently high levels of EtOH consumption (Dole and Gentry, 1984; Rhodes et al., 2005; Yoneyama et al., 2008).

## Behavioral And Physiological Outcomes by Diet, Alcohol, and the Combination

### Changes by Diet

Numerous behavioral and physiological changes have been positively linked to obesity in both clinical and preclinical studies. In clinical studies (see Robinson et al., 2020 for recent review), there is highly suggestive evidence that heavier body weight is associated with impaired executive functioning, increased impulsivity, and impaired reward-related decision making, as well as suggestive evidence of impaired self-esteem and body image concerns. The most prevalent clinical data show that heavier body weight increases the likelihood of experiencing anxiety and depression (Bonnet et al., 2005; Petry et al., 2008; Gariepy et al., 2010; Preiss et al., 2013; Robinson et al., 2020). Preclinical studies investigating anxiety- and depressive-like behaviors also show an increase in these measures in HFD-fed rodents (Sivanathan et al., 2015; Zemdegs et al., 2016; Gelineau et al., 2017). The well-known physiological consequences of obesity in the clinical population, such as insulin resistance, glucose intolerance, and increased risk for developing type II diabetes (Haslam and James, 2005), are recapitulated in rodent models. Animal studies show that both short-term and chronic HFD consumption results in glucose intolerance and insulin resistance as well as increased fasting glucose, insulin, free fatty acid, triglyceride, and leptin levels compared to regular chow diet (Hariri and Thibault, 2010; Paulson et al., 2010; Dutheil et al., 2016; Coker et al., 2020). Female mice are often shown as being more resistant to these HFD-induced physiological changes, potentially due to metabolically protective effects of estrogen (Gelineau et al., 2017). Consistent with this, a study done in female rats showed that metabolic disturbances induced by loss of estrogen due to ovariectomy (i.e., increase in weight gain, hyperleptinemia, glucose intolerance) were exacerbated by HFD. Induction of depressive-like behaviors accompanied metabolic deterioration in these rats indicating that HFD may increase vulnerability to development of depression in the absence of estrogen (Boldarine et al., 2019). It is important to recognize, however, that not all studies show that females are resistant to metabolic disturbances (White et al., 2019) and that sex as a biological variable in HFD-induced changes in metabolic function and depression or anxiety-like behavior remains to be fully elucidated. It is also important to note that diet-induced behavioral alterations, assessed with forced swim test and elevated plus maze test, can be due to many factors, including length of diet exposure. For instance, HFD in rats negatively impacted brain homeostasis and inflammation by disrupting intracellular cascades involved in synaptic plasticity, insulin signaling, and glucose homeostasis and by increasing corticosterone levels and inflammatory cytokines and was related to anxiety- and anhedonia-like behaviors (Dutheil et al., 2016). In this same study, it was demonstrated that the consequences of HFD might be contingent on duration of exposure. While no effect was seen after 8 weeks of exposure, HFD given for 16 weeks in rats resulted in significant differences in behavioral measures of anxiety (assessed using novelty suppressed feeding test, open field test, elevated plus maze test, and novel object recognition test) and anhedonia (determined using sucrose preference test and female urine sniffing test) (Dutheil et al., 2016). Overall, the impact of HFD and other obesogenic diets on anxiety, depression and other behaviors remains in important area of research.

### Changes by Alcohol

The comorbidity between EtOH consumption and anxiety has been well-studied in both clinical (Kushner et al., 2000) and preclinical (Pandey et al., 2005; Crews et al., 2016) populations across the entire lifespan. Depressive disorders are also commonly comorbid psychological conditions in people with AUD (Grant et al., 2004), with their co-occurrence leading to increased severity of symptoms (Hasin et al., 2002). Epidemiologic data on the physiological consequences of EtOH intake differ depending on intake patterns. Moderate EtOH consumption appears protective against insulin resistance in both clinical (Davies et al., 2002; Koppes et al., 2005; Yokoyama, 2011; Bonnet et al., 2012; Traversy and Chaput, 2015) and preclinical (Paulson et al., 2010) studies, while acute and chronic/binge consumption is associated with insulin resistance in non-obese humans (Fan et al., 2006; Papachristou et al., 2006; Ting and Lautt, 2006) and rodents (Dhillon et al., 1996; Lindtner et al., 2013). As a result, the relationship between EtOH consumption and insulin sensitivity is often described as an inverted U-shape (Villegas et al., 2004; Koppes et al., 2005; Ting and Lautt, 2006).

The effects of EtOH on glucose tolerance and leptin levels in healthy humans and rodents are also inconsistent. Standard oral glucose tolerance tests in the clinical population have shown no change (Singh et al., 1988; Beulens et al., 2008) or improvement (McMonagle and Felig, 1975) in glucose tolerance following moderate EtOH consumption, and impaired glucose tolerance in subjects who have chronically consumed EtOH (Andersen et al., 1983). Animal studies show moderate EtOH consumption can lead to no change (Hong et al., 2009; Paulson et al., 2010; Gelineau et al., 2017) or improvement (Hong et al., 2010) in glucose tolerance, while chronic consumption promotes glucose intolerance (Feng et al., 2010) in mice and rats. Additionally, chronic EtOH consumption has been shown to elevate (Nicolas et al., 2001; Obradovic and Meadows, 2002) or decrease (Hiney et al., 1999) leptin levels in rodent models, while moderate consumption produced no change in mice and humans (Beulens et al., 2008; Gelineau et al., 2017). Overall, the discrepancies on the impact of EtOH consumption on metabolic parameters appear to be due to differences in patterns of EtOH intake or percentage of EtOH in solution used. This can make interpretation of results across studies difficult and suggests standardized models of EtOH effects on metabolic and other physiological functions may be important in future studies.

### Changes by Combination of Diet and Alcohol

Chronic consumption of both HFD and EtOH likely has combinatorial effects on overall mental and physiological health. For instance, patients diagnosed with anxiety or depression are more likely to be obese and to binge drink (Strine et al., 2008). Preclinical studies show that concurrent EtOH and HFD consumption increases anxiety measures in the light/dark box in predominantly in female mice (Gelineau et al., 2017). The epidemiologic evidence described in the previous sections for potential beneficial physiological effects of moderate EtOH consumption on insulin sensitivity may only occur in non-obese patients (Yokoyama, 2011). Most preclinical studies, however, show that moderate EtOH consumption mitigates HFD-induced metabolic dysfunction (Hong et al., 2009; Paulson et al., 2010; Gelineau et al., 2017). These studies, specifically, show that moderate EtOH consumption improves insulin sensitivity and glucose tolerance in mice on HFD, without affecting HFD induced changes in body mass or circulating insulin and leptin levels. As discussed above, many of the discrepancies in the HFD and EtOH co-consumption literature may be due to differences in methodology of consumption between studies. To address this possibility, studies by our lab (Coker et al., 2020) show that differences in the scheduling of EtOH and HFD access mediate the amount of EtOH consumed and the resultant impact on insulin and glucose function. This study showed that moderate and binge EtOH consumption does not improve insulin sensitivity or glucose tolerance in HFD-fed mice. Additionally, binge consumption of both HFD and EtOH promoted insulin resistance and glucose intolerance in the absence of an overweight phenotype. Clinically, such metabolic dysregulation in lean individuals is linked to increased risk for more severe type II diabetes as well as increased total and cardiovascular-related mortality compared with overweight diabetic individuals (George et al., 2015; Olaogun et al., 2020). Thus, there may be an unrecognized clinical population that may be seemingly healthy due to overall lower BMI but have underlying metabolic dysfunction that may not be fully considered. Overall, these findings suggest there are various factors that may affect how EtOH and HFD interact to influence metabolic disturbances, such as frequency and duration of access. Standardization of HFD and EtOH co-consumption models may improve upon discrepancies in the field.

## Potential Central Mechanisms of High Fat Diet and Alcohol Behavioral Interactions

### Clinical and Preclinical Studies of Mechanisms of High Fat Diet Modulation of Central Neurocircuits

Two systems interact in the regulation of feeding behavior – homeostatic systems and brain reward systems (Kenny, 2011a). The homeostatic system involves metabolic mechanisms including hormonal regulators from the periphery (e.g., ghrelin, insulin, leptin) that control hunger, satiety, and adiposity. Energy balance is maintained at homeostatic levels by these hormones acting on various hypothalamic (paraventricular nucleus, PVN; arcuate nucleus, ARC; lateral nucleus; dorsomedial nucleus) and hindbrain (nucleus of the tractus solitarius; raphe nucleus) feeding circuits. Research on the integrative signaling of homeostatic and hedonic feeding in the central nervous system (CNS) suggests that endocrine signals from the periphery, such as hormonal regulators of appetite, can act on other CNS regions outside of the traditionally studied hypothalamic and brainstem regions, such as dopaminergic (ventral tegmental area, VTA) and limbic (amygdala, hippocampus, cortex) regions (Stice et al., 2013). While these two systems interact to influence food intake, it has been shown that the hedonic properties of food can override the homeostatic system to stimulate feeding behavior resulting in hyperphagia even after energy requirements have been met. Consequently, stimulation of the reward systems to promote overconsumption of palatable food can lead to overweight and obesity (Kenny, 2011a,b). The HFD-induced sensitization of these pathways does not appear to normalize following return to standard diet in rodent models (Mazzone et al., 2020), suggesting long term consequences to consummatory pathways is not easily resolved and may trigger compulsive eating behaviors (Moore et al., 2017a,b). For more detailed information on these well-defined neurocircuits, we direct readers to the following reviews that outline how appetite and energy homeostasis are regulated by various hypothalamic circuits (Cassidy and Tong, 2017; Sternson and Eiselt, 2017; Timper and Brüning, 2017; Chowen et al., 2019) and reviews that focus on the different subdivisions of the amygdala and reward (Baxter and Murray, 2002; Gilpin et al., 2015; Janak and Tye, 2015; Wassum and Izquierdo, 2015; Daviu et al., 2019).

### Clinical and Preclinical Studies of Mechanisms of Alcohol Use Disorders Across the Lifespan

In addition to highly prevalent AUD risk in adult populations, EtOH is the most commonly used substance of abuse among adolescents and is a major public health concern in the United States^4^. Underage drinking makes up 11% of all EtOH consumed nationwide and over 90% of this is in the form of binge drinking^5^. The adolescent brain is particularly vulnerable to many effects of EtOH (Guerri et al., 2009) and there are countless long-lasting negative consequences associated with underage binge drinking, including an increased risk for developing alcohol/substance use disorders (Grant and Dawson, 1997; DeWit et al., 2000; Guerri and Pascual, 2016) and other mental health issues later in life (Spear, 2016). Animal models recapitulate many of the findings of the clinical literature, suggesting long lasting perturbations in neurobiological functions following adolescent EtOH exposure in rodents (Hiller-Sturmhöfel and Spear, 2018; Spear, 2018; Crews et al., 2019) that lead to increased susceptibility to EtOH effects in adulthood. For instance, it has been shown that intermittent EtOH exposure in adolescent rats, as a model for binge drinking behaviors, produces anxiety-like behaviors and is associated with increased EtOH consumption in adulthood (Pandey et al., 2015). Preclinical studies exploring activity of the mesocorticolimbic dopamine reward system in response to adolescent EtOH consumption report an increase in dopamine neurotransmission compared to EtOH-naïve rats (Sahr et al., 2004), as well as an increase in extracellular dopamine levels in the nucleus accumbens (NAc) of EtOH-treated adolescent rats compared to EtOH-treated adult rats (Pascual et al., 2009) which can continue into adulthood (Badanich et al., 2007). Remodeling changes in the neural circuits that make up the mesocorticolimbic pathway and alterations in dopamine function in these circuits occur throughout adolescence (Spear, 2000). Thus, it is plausible that sensitization of the mesocorticolimbic dopaminergic pathway following adolescent EtOH exposure mediates the long-term susceptibility of developing AUD later in life, although many other pathways have been suggested to be involved in this phenomenon (Crews et al., 2019).

The likelihood of developing AUD is not limited to early onset of EtOH consumption. Chronic and binge-like EtOH exposures can increase subsequent EtOH intake in adult clinical research and in preclinical models, suggesting a gradual transition from social drinking to excessive EtOH consumption and diagnosis of AUD can be modeled preclinically. This progressive transition has successfully been simulated in rodent models of intermittent access to EtOH in a two-bottle choice paradigm (Carnicella et al., 2014). For example, intermittent access to EtOH in rats has been shown to escalate subsequent self-administration of EtOH (those in a post-dependent state) compared to those given continuous access to EtOH or with no history of EtOH (Kimbrough et al., 2017). Additionally, the increase in self-administration of EtOH observed in rats with a history of EtOH dependence induction persists even after weeks of withdrawal (Roberts et al., 2000). This model of EtOH dependence may be due to allostatic changes in reward function due to modifications in neurotransmitter systems known to be involved in regulating the neurobiology of the positive reinforcing effects of EtOH (Koob, 2003).

The compulsive-like drug seeking effect associated with AUD is thought to involve, among other mechanisms, corticotropin-releasing factor (CRF) signaling in the extended amygdala (i.e., central amygdala, CeA and bed nucleus of the stria terminalis, BNST), which mediates the negative affective states typical of EtOH withdrawal and post-abstinence craving behaviors (Koob and Volkow, 2016; Zorrilla and Koob, 2019). CRF release increases within the CeA and BNST of EtOH dependent rats during withdrawal (Olive et al., 2002; Funk et al., 2006), which is thought to drive the increase in subsequent self-administration of EtOH. Studies indicating the CRF system is critical for the increase in self-administration in the post-dependent state show that treatment with CRF receptor antagonists reduces subsequent EtOH self-administration in these animals, specifically when injected in the CeA (Funk et al., 2006), with no effect in non-dependent animals (Funk et al., 2007).

### Clinical and Preclinical Studies of High Fat Diet-Induced Alcohol Intake

Numerous clinical studies show increased desire, cravings, and intake of high fat foods during and after EtOH drinking episodes (Caton et al., 2004; Breslow et al., 2013; Piazza-Gardner and Barry, 2014). Epidemiologic data report 433 kcal excess consumption in men on drinking days vs. non-drinking days but only 61% of the excess calories is made up by the EtOH itself (Breslow et al., 2013), suggesting EtOH intake can drive increased food intake. Findings also suggest the effect of EtOH on food intake is modulated by increasing appetite and delaying the sensation of satiety (Caton et al., 2004). Less is known clinically, however, about the potential for HFD consumption to increase EtOH intake. Cross-sectional studies report that binge eating behaviors, which can be associated with increased propensity for obesity, are not only associated with increased intake of food higher in energy density, but also increased consumption of EtOH (Muñoz-Pareja et al., 2013; Bogusz et al., 2021). There are many shared mechanisms between binge eating disorders and drug addiction, including alterations in reward signaling and emotional processing (Carlier et al., 2015; Schulte et al., 2016; Moore et al., 2017a,b), which suggests that binging may be part of an overall compulsive behavioral phenotype that can straddle reward modalities. These findings may also suggest, therefore, that binge HFD intake may be a risk factor for escalation of EtOH intake. In the preclinical setting, a similar positive relationship for EtOH-induced increases in HFD intake has been shown in some animal models (Barson et al., 2009). While the inverse relationship of acute HFD exposure stimulating EtOH intake has been suggested in some animal models (Carrillo et al., 2004), the majority of findings indicate that HFD access decreases EtOH consumption in rodent models (Feng et al., 2012; Gelineau et al., 2017; Sirohi et al., 2017a,b). These discrepancies may be due, at least in part, to differences in HFD access periods.

Our recent work indicates that an intermittent HFD model using repeated acute HFD access periods once per week and intermittent, limited access to EtOH on days HFD is not given significantly increases EtOH intake in mice compared to both continuous control and HFD diet access (Coker et al., 2020). This is in contrast to other studies that show intermittent high fat/high sucrose diets given twice a week or more reduce EtOH intake in rats (Sirohi et al., 2017b). Additional data from our lab suggest that multiple HFD access days per week reduces EtOH intake and preference in mice at lower concentrations (10%), but this reduced intake and preference is not observed at higher EtOH concentrations (20%) compared to mice given HFD access only one time per week. These findings suggest that HFD may alter the overall reward value of EtOH, which is dependent on how many HFD access periods are available. Overall, it appears that access schedules mediate the interaction between binge HFD and binge EtOH intake in preclinical models. These differences also appear to have clinical relevance that may distinguish the ability of diets to induce binge eating disorders, substance use disorders, and their combination. These data, along with the findings discussed above showing palatable diets may decrease EtOH intake under certain conditions, suggest that dietary modifications during treatment for alcohol or drug use disorders may be an important avenue for future research to improve both behavioral and metabolic clinical outcomes.

### Central Mechanisms of High Fat Diet and Alcohol Overlap

As described in previous sections, emerging evidence suggests similarities in the regulation of the overconsumption of palatable diets and drugs of abuse. For example, a review by Barson et al. (2011) focused on the similarities in hypothalamic and mesocorticolimbic circuits which act to regulate the intake of both food and EtOH. Interestingly, feeding research has historically focused on the hypothalamus, as its various nuclei are known to modulate food intake. The focus of feeding research has now expanded to include mesocorticolimbic regions that also regulate hedonic food intake. Specifically, studies show an increase in dopamine within the NAc while feeding or in the presence of food, an effect that lasted after consumption ceased. This increase was observed in mice made underweight but fed prior to the study as well as in mice mildly food deprived and trained to bar press for food (Hernandez and Hoebel, 1988; Smith and Schneider, 1988). Although it has long been recognized that EtOH can impact hypothalamic circuits regulating feeding behaviors (Amit et al., 1975), EtOH research has historically examined mesolimbic dopaminergic and other emotional regulatory circuits such as the extended amygdala. There is renewed interest in recent years, however, regarding EtOH effects on neuropeptide systems typically examined in food intake pathways (Leibowitz, 2007; Olney et al., 2014; Carlier et al., 2015; Alhadeff et al., 2019). Additionally, there are neuronal networks that extend between hypothalamic and mesocorticolimbic regions that may regulate the consumption of palatable food and drugs of abuse. Hypothalamic neurons can project to other hypothalamic subregions as well as to extra-hypothalamic regions, such as from the PVN to the VTA (Rodaros et al., 2007). Projections from the lateral hypothalamus extend to amygdala, NAc, VTA, and prefrontal cortex, and these regions also project back to the hypothalamus (Beckstead et al., 1979; Kita and Oomura, 1981; Fadel and Deutch, 2002; Kampe et al., 2009). Interestingly, aside from overlapping neurocircuit and neurochemical systems, endocrine signals from the periphery involved in the homeostatic control of feeding (e.g., leptin, ghrelin, insulin) also have direct effects on dopaminergic function and the reward value of food (Stice et al., 2013). Studies show satiety hormones, such as leptin and insulin, decrease food reward, dopamine release, and dopamine neuronal excitability (Figlewicz et al., 2004, 2006; Figlewicz and Benoit, 2009; Mebel et al., 2012), whereas the hunger hormone ghrelin increases food reward and dopaminergic function (Overduin et al., 2012; PerellóPerelló and Zigman, 2012). Overall, these findings suggest multiple overlapping connections between feeding and reward circuitry.

The overlap in hedonic intake mechanisms between both palatable food and drugs of abuse suggests overconsumption of these two reinforcers may share common mechanisms contributing to central dysfunction. The mesolimbic dopamine reward system, which projects from the VTA to the NAc and other limbic regions, is a key focus when studying the overlapping neurobiology of substance misuse and palatable diet overconsumption (Stice et al., 2013; Volkow et al., 2017). Studies show that consumption of both palatable food and EtOH stimulate dopaminergic neuron firing in the VTA and dopamine release in the NAc and prefrontal cortex (Yoshida et al., 1992; Wilson et al., 1995; Martel and Fantino, 1996; Volkow et al., 2002; Gambarana et al., 2003; Yan et al., 2005; Liang et al., 2006; Morzorati et al., 2010; Ding et al., 2011). The role of insulin on dopaminergic neurons within various mesolimbic brain regions is noteworthy. In the VTA, insulin has been shown to decrease dopaminergic neuron firing (Labouèbe et al., 2013) and is thought to aid in the termination of food intake (along with homeostatic signaling in the hypothalamus), whereas in the NAc insulin potentiates release and reuptake at dopamine terminals and is thought to have influence on regulation of the rewarding characteristics of food intake (Stouffer et al., 2015). Studies show that HFD-induced insulin resistance alters dopamine terminal function in the NAc, which is reversed by restoring insulin signaling (Fordahl and Jones, 2016), suggesting central insulin resistance after HFD exposure may be an important factor in hedonic food intake and impaired satiety.

The amygdala, a limbic region that acts as an integrative center for emotions, memory, and motivation, also regulates food intake and reward. Clinical studies using functional magnetic resonance imaging (fMRI) show an increase in amygdala activity in overweight and obese adults (Ho et al., 2012) and children (Boutelle et al., 2015) in response to visual food cues and appetitive taste even in the postprandial state, indicating impaired satiety. Early preclinical studies investigating the role of the amygdala on feeding behavior indicate hyperphagia, increased weight gain, and hyperinsulinemia following bilateral lesions of the amygdala (King et al., 1994, 1996). Furthermore, studies show an increase in dopamine turnover in the amygdala following food intake in lean rats, which in turn is involved in reducing food intake (Anderberg et al., 2014). The anorexigenic hormone, glucagon-like peptide 1 (GLP1), was also shown to increase dopamine transmission in these animals and consequently reduce food intake (Anderberg et al., 2014). Thus, dopamine signaling is thought to be an important mechanism behind the regulation of food intake in the amygdala. Interestingly, GLP1 receptor agonists have recently been utilized in preclinical models to reduce reinstatement of drug seeking behaviors (Douton et al., 2020), suggesting drug targets that can improve metabolic outcomes in obesity (Iepsen et al., 2020) may be useful in the treatment of substance use disorders. Additionally, insulin receptor signaling in the amygdala is similar to that of the hypothalamus, which results in reduced food intake. This signaling is disrupted in rodents on an obesogenic diet resulting in insulin resistance in the amygdala (Areias and Prada, 2015). Such HFD-induced disruptions to amygdala signaling may enhance anxiety- and depressive-like behaviors in rodent models and potentially in the clinical population. The aforementioned findings suggest that insulin and other anorexigenic hormones may engage multiple mechanisms to promote satiety and influence reward salience, specifically by acting on various brain regions including the mesolimbic dopamine reward system, amygdala, and hypothalamus.

Recent evidence has identified additional overlapping brain regions, such as the nucleus of the tractus solitaries (NTS), that are involved in reward processing of palatable food and drugs of abuse (Kenny, 2011b). The NTS is well-known for receiving afferent information from the gastrointestinal tract triggered by gastric distension following ingestion of food (Garcia-Diaz et al., 1988), then projects to the hypothalamus sending satiety signals which collectively makes up the homeostatic regulation of feeding behavior. The NTS also receives afferent information from chemosensory neurons in the oral cavity that detect the palatability of food. All incoming information is then processed by various neuronal populations in the NTS, such as those expressing tyrosine hydroxylase (TH), pro-opiomelanocortin (POMC), and GLP1 (Sumal et al., 1983; Appleyard et al., 2005; Hayes et al., 2009). These neurons in turn project to various brain regions involved in detecting the hedonic properties of food and drugs of abuse, including limbic regions involved in reward processing (i.e., NAc, CeA, BNST), hypothalamic and thalamic regions, and prefrontal cortex. These numerous central and peripheral systems interact to control food and drug intake and may act as locations for sensitization of rewarding properties of HFD and EtOH or other drugs.

While this review focuses on the shared central mechanisms between binge eating disorders and drug addiction, suggesting the possibility binge eating disorders might represent an addiction-like behavioral state toward food, it is important to discuss criticism the theory of food addiction has received. Binge eating has been conceptualized as a form of addictive behavior due to overlapping properties shared with other accepted addictive behaviors (i.e., AUD), such as the loss of control over consumption and repeated engagement in binge behaviors despite negative consequences (Ferriter and Ray, 2011), as well as overlapping symptoms and genetic factors (Davis, 2013, 2017; Munn-Chernoff and Baker, 2016). A review by Rogers (2017) meticulously outlines the similarities and differences in appetites for foods and drugs. While there are clearly many similarities, one important difference between food and drugs of abuse is that food is necessary for survival while drugs of abuse are not. Furthermore, drugs of abuse are more potent reward signals than food in general, which likely leads to stronger levels of plasticity in brain circuitry controlling intake. Rogers concludes that while binge eating disorders may fulfill some key criteria of addictive behavior, broadening the definition of addiction to include food intake in general is likely detrimental to the impact of current research to both binge eating disorders and substance use disorders. Therefore, while there are many similarities between binge eating and binge drug and alcohol intake, including overlap of neurocircuitry and the potential for cross-sensitization of binge behaviors across modalities described above, “food addiction” as a generalized term is inaccurate and does not fully explain the continued high prevalence of overweight and obesity (Rogers, 2017).

## Neuroimmune Function

Overall, the shared neurobiology of addiction and obesity may be attributed to common brain regions and neurotransmitter systems involved in the regulation of consumption of palatable food and drugs of abuse, suggesting overlapping central dysfunction may be involved. The effects of palatable diet and drugs of abuse on reward circuits, such as the mesolimbic dopamine reward system, may be a key focus of this overlap. The influence of peripheral hormones on these reward systems are also noteworthy. As discussed in this section, one potential mechanism for the increase of disease susceptibility (i.e., diet-induced obesity and alcoholism) involving these brain regions may be neuroimmune function.

### High Fat Diet Effects on Hypothalamic Neuroinflammation/Microglia

Microglia are the resident immune cells of the brain and play a key role in HFD-induced neuroinflammation. “Resting” or “ramified” microglia mainly exist under non-pathological conditions with their highly branched processes surveying the CNS. Upon stimulation, including detection of neuronal damage or systemic inflammation, microglia undergo morphological changes and rapidly convert from the resting state to the more amoeboid, non-ramified “active” state allowing for phagocytosis of pathogens or cellular debris (Kreutzberg, 1996; Ransohoff and Cardona, 2010; Cerbai et al., 2012; Salter and Beggs, 2014). Preclinical studies show hypothalamic inflammation following HFD is marked by a rapid increase in microglia activity which is thought to be a critical regulator of susceptibility to DIO (Valdearcos et al., 2017).

It is well-characterized that HFD exposure is closely associated with enhanced peripheral and central inflammation and this is a common feature of obesity, insulin resistance, and type II diabetes. However, it is now been shown that neuroinflammation due to HFD consumption can precede the onset of body mass increases or peripheral inflammation in animal models. Hypothalamic insulin resistance appears to be an antecedent to peripheral insulin resistance, as the development of inflammatory responses and insulin resistance to HFD occurs much more rapidly in this brain region compared to peripheral tissues (Prada et al., 2005; Valdearcos et al., 2015). Rodent studies show that even a single day of HFD exposure induces neuroinflammation in the hypothalamus (Waise et al., 2015). This process occurs before an increase in adiposity or peripheral inflammation, suggesting increased body mass is not required for development of insulin resistance in the hypothalamus (Rorato et al., 2017). Importantly, long-lasting changes in hypothalamic neuroinflammation occur in rodent models of developmental HFD exposure, leading to persistent changes in peripheral metabolic function independent of body mass (Cai and Liu, 2011). Together, these findings indicate that neuroinflammation in the hypothalamus may be a key driver to both central and peripheral alterations in insulin receptor function and metabolic outcomes following HFD intake.

### High Fat Diet Effects on Extra-Hypothalamic Neuroinflammation

HFD-induced neuroinflammation has typically been studied in the hypothalamus, due to its distinct neuronal populations that are specific to regulating food intake and energy expenditure. Neuroinflammation brought on by various obesogenic diets has also been observed in multiple brain regions such as the amygdala, hippocampus, and cerebellum (Guillemot-Legris and Muccioli, 2017). Studies show that HFD-induced obesity increases expression of the pro-inflammatory cytokine interleukin-1 beta (IL-1β) (Almeida-Suhett et al., 2017) and induces insulin resistance in the amygdala through increased activation of the pro-inflammatory transcription factor nuclear factor-kappa B (NF-κB) (Castro et al., 2013). Animal studies indicate enhanced NF-κB inflammatory signaling (Lu et al., 2011; Kang et al., 2016), increased inflammatory markers (i.e., IL-1β, IL-6, and TNFα) (Lu et al., 2011; Miao et al., 2013), and heightened glial activation (i.e., astrogliosis and microgliosis) (Lu et al., 2011; Rivera et al., 2013; Kang et al., 2016) in the hippocampus following HFD exposure. In the mesolimbic reward pathway of non-human primates, emerging evidence indicates an obesogenic diet alters dopamine signaling and functional connectivity between the NAc and prefrontal cortex related to an increase in inflammatory markers (Godfrey et al., 2020). Thus, diet-induced inflammatory signaling may alter dopaminergic reward systems, increasing HFD intake, and potentially modulate intake of other rewards, such as drugs of abuse. Increased adiposity in DIO models may also lead to sensitization of stress mediated pro-inflammatory cytokine release from adipose cells (Qing et al., 2020), potentially leading to a feed-forward mechanism for enhanced central dysfunction in reward, stress, and metabolic regulatory neurocircuitry.

### Alcohol Effects on Neuroinflammation/Microglia

EtOH consumption leads to a host of complications involving the CNS including inflammation and impairment of neuroimmune responses. A single dose of EtOH (5 g/kg intraperitoneal) has been shown to increase pro-inflammatory mRNA expression in mouse brains (Qin et al., 2008). Microglia are becoming recognized as critical modulators of EtOH-induced neurotoxicity (Henriques et al., 2018; Melbourne et al., 2019) since there is an increase in their activation markers in post-mortem brains of human alcoholics (He and Crews, 2008). Research shows there are alterations in CNS innate immune signaling upon exposure to EtOH, which increases activation of microglia and in turn exacerbates the neurotoxicity of EtOH. As a result, this EtOH-induced microglia pro-inflammatory activation leads to increased production of inflammatory cytokines, such as IL-1β, IL-6, and TNFα through the activation of the pro-inflammatory transcription factor NF-κB (Zou and Crews, 2010, 2012; Zhao et al., 2013; Hernandez et al., 2016). Excessive production of these cytokines and other pro-inflammatory factors, such as reactive oxygen species (ROS), by activated microglia contribute to EtOH-related pathologies (Alfonso-Loeches and Guerri, 2011).

The extent of the aforementioned effects of EtOH on neuroimmune responses and neurotoxicity by microglia activity differ depending on length of EtOH exposure, age of exposure, and brain region examined (Chastain and Sarkar, 2014; Perkins et al., 2019). Studies report microglia are partially activated following binge EtOH exposure which may cause increased production of anti-inflammatory factors (Marshall et al., 2013), whereas chronic intermittent EtOH exposure fully activates microglia which increases production of pro-inflammatory cytokines and ROS thereby resulting in neurotoxicity (Qin and Crews, 2012a,b; Zhao et al., 2013). These data align with the concept of microglial activation being a graded response resulting in various active microglial phenotypes (Melbourne et al., 2019).

EtOH-induced microglia activation is also considered in the development of AUD through microglia priming. It is hypothesized that adult chronic EtOH exposure can sensitize the immune system, specifically microglia, long-term thus increasing the risk for developing EtOH-related disorders (Chastain and Sarkar, 2014). Similar long-term immune sensitization is seen following prenatal EtOH exposure. This hypothesis is supported by findings of a greater increase in microglia activation in response to a systemic immune challenge in mice with a history of chronic EtOH (Qin and Crews, 2012a) and a study that reported prenatal EtOH exposure had a long-term effect on immune function in rats signified by increased severity of inflammation in a rheumatoid arthritis animal model (Zhang et al., 2011). Additionally, studies show that EtOH-induced neuroinflammation (measured by microglia activation) is not reversed by long-term withdrawal (Cruz et al., 2017), indicating long-term microglia sensitization.

Interestingly, EtOH administration in binge-like patterns induces whole-body insulin resistance and disruptions in glucose homeostasis by impairing hypothalamic insulin receptor function (Lindtner et al., 2013). These findings suggest that effects of EtOH on hypothalamic neuroimmune function are also critical for modulation of insulin and glucose homeostasis, similar to effects of HFD on hypothalamic neuroimmune function. Overall, these studies indicate that neuroimmune signaling and function may be a key point of cross-sensitization between HFD and EtOH to regulate aspects of peripheral and central metabolic function as well as emotional processing behaviors. Future studies will be needed to directly examine this possibility.

## Summary

The increased prevalence of obesity, AUD, and psychiatric conditions such as anxiety and depression in the United States has become a major public health concern. The shared clinical consequences of chronic and binge HFD intake and chronic EtOH misuse suggest an overlap in the mechanisms by which these factors modulate whole body physiology and central pathologies related to emotional regulation pathways indicated in psychiatric conditions. While we present evidence that HFD and EtOH intake may cross-sensitize binge behaviors and metabolic consequences via overlapping central mechanisms, a better mechanistic understanding of these processes may allow alteration of diets to be of benefit during AUD treatment, as has been suggested in other studies (Brutman et al., 2020; Shah et al., 2020). An important topic not discussed in this review is the effect of HFD and EtOH on microbiome gut-brain interactions. Since research is just beginning to understand the important roles of the gut microbiome on HFD-induced metabolic dysregulation and in EtOH-related behaviors individually, it is too early to properly assess how the gut microbiome might be impacted by a combination of HFD and EtOH intake. We refer readers to these other reviews that have discussed aspects of the gut microbiome on regulation of metabolic function, neurocircuits, and behaviors (e.g., Schellekens et al., 2012; Torres-Fuentes et al., 2017; Jerlhag, 2019; Cryan et al., 2020). In summary, we highlight numerous potential mechanisms behind cross-sensitization of HFD and EtOH effects in both behaviors and metabolic function, including alterations in neurotransmitter systems and neuroimmune function in shared neurocircuitry regulating emotional processing and peripheral signaling that will be important for future investigations.

## Author Contributions

All authors wrote and edited the manuscript.

## Conflict of Interest

The authors declare that the research was conducted in the absence of any commercial or financial relationships that could be construed as a potential conflict of interest.

## Footnotes

^1^(1998). Executive summary. *Obes. Res*. 6, 51S-179S. doi: 10.1002/j.1550-8528.1998.tb00690.x

^2^CDC National Health Report Highlights *CS*251163.

^3^Section 5 PE Tables – Results from the 2018 National Survey on Drug Use and Health: Detailed Tables, Sections 1–3, SAMHSA, CBHSQ. Available online at: https://www.samhsa.gov/data/sites/default/files/cbhsq-reports/NSDUHDetailedTabs2018R2/NSDUHDetTabsSect5pe2018.htm#tab5-4a (accessed March 30, 2020).

^4^*Underage Drinking | National Institute on Alcohol Abuse and Alcoholism (NIAAA)*. Available online at: https://www.niaaa.nih.gov/publications/brochures-and-fact-sheets/underage-drinking (accessed June 16, 2020)

^5^Underage Drinking | CDC. Available online at: https://www.cdc.gov/alcohol/fact-sheets/underage-drinking.htm (accessed June 16, 2020)
